# Emergence of vancomycin resistant *Staphylococcus aureus *(VRSA) from a tertiary care hospital from northern part of India

**DOI:** 10.1186/1471-2334-6-156

**Published:** 2006-10-26

**Authors:** Hare Krishna Tiwari, Malay Ranjan Sen

**Affiliations:** 1 Department of Microbiology, Institute of Medical Sciences, Banaras Hindu University, Varanasi-221005, India

## Abstract

**Background:**

Glycopeptides such as vancomycin are frequently the antibiotics of choice for the treatment of infections caused by methicillin resistant *Staphylococcus aureus *(MRSA). For the last 7 years incidence of vancomycin intermediate *S. aureus *and vancomycin resistant *S. aureus *(VISA and VRSA respectively) has been increasing in various parts of the world. The present study was carried out to find out the presence of VISA and VRSA in the northern part of India.

**Methods:**

A total 1681 staphylococcal isolates consisting of 783 *S. aureus *and 898 coagulase negative staphylococci (CoNS) were isolated from different clinical specimens from various outpatient departments and wards. All *S. aureus *and 93 CoNS were subjected to MIC testing (against vancomycin, teicolplanin and oxacillin); Brain Heart Infusion (BHI) vancomycin screen agar test; disc diffusion testing, and PCR for *mecA*, *vanA *and *vanB *genes detection.

**Results:**

Out of 783 *S. aureus *two *S. aureus *strains were found to be vancomycin and teicoplanin resistant (one strain with MIC 32 μg/ml and the other strain with MIC 64 μg/ml); six strains of *S. aureus *have shown to be vancomycin intermediate (two strains with MIC 16 μg/ml and four strains with MIC 8 μg/ml); and two strains with teicoplanin intermediate (MIC 16 μg/ml). One CoNS strain was resistant to vancomycin and teicoplanin (MIC 32 μg/ml), and two CoNS strains were intermediate to vancomycin and teicoplanin (MIC 16 μg/ml). All VRSA, VISA and vancomycin resistant CoNS had shown growth on BHI vancomycin screen agar (vancomycin 6 μg/ml) and were *mecA *PCR positive. None of these isolates have demonstrated *vanA/vanB *gene by PCR.

**Conclusion:**

The present study reveals for the first time emergence of VISA/VRSA from this part of world and indicates the magnitude of antibiotic resistance in and around the study area. The major cause of this may be unawareness and indiscriminate use of broad-spectrum antibiotics.

## Background

*Staphylococcus aureus *has long been recognized as a major pathogen of hospital acquired infections. Over the last decade, methicillin resistant *S. aureus *(MRSA) strains have become endemic in hospitals worldwide. In addition, it is now incipient community pathogen in many geographical regions [1]. MRSA is important because, in addition to being methicillin resistant, most strains are also resistant to other β-lactam antibiotic, with the exception of glycopeptide antibiotics [2,3]. In 1980s, because of widespread occurrence of MRSA, empiric therapy for staphylococcal infections (particularly nosocomial sepsis) was changed to vancomycin in many health care institutions. Vancomycin use in United States also increased during this period because of the growing numbers of infections with *Clostridium difficile *and coagulase negative staphylococci (CoNS) in health care institutions [4,5]. Thus, the early 1990s have shown a discernible increase in vancomycin use. As a consequence, selective pressure was established that eventually lead to the emergence of strains of *S. aureus *and other species of staphylococci with decreased susceptibility to vancomycin and other glycopeptides [6]. In 1997, the first strain of *S. aureus *with reduced susceptibility to vancomycin and teicoplanin was reported from Japan [7]. Shortly after, two additional cases were reported from United States [8]. However, first clinical isolate of vancomycin resistant *S. aureus *(VRSA) was reported from United States in 2002 [9]. More recently some workers have reported vancomycin resistant staphylococcal stains from Brazil [10] and Jordan [11].

Strains of Vancomycin Intermediate *S. aureus *(VISA) with vancomycin MIC of 8 μg/ml have been reported from Japan [7], United States [12-14], France [15], United Kingdom [16] and Germany [17]. Most of these isolates appear to have developed from preexisting MRSA infections. Assadullah et al from India have reported reduced susceptibility of vancomycin against MRSA and CoNS [18]. Keeping this in view we performed extensive longitudinal study of total 1681 staphylococcal strains for the assessment of current situation of vancomycin resistance in northern part of India. This is the first report of VRSA emergence from a tertiary care hospital from this part of world.

## Methods

### Staphylococcal Isolates

A total 1681 staphylococcal isolates consisting of 783 *S. aureus *and 898 CoNS were investigated for the period of three years from August 2002 to July 2005. The strains were collected from various clinical specimens including pus, urine, wound swabs, catheters, blood, sputum and CSF from the patients of different inpatient and outpatient departments of the University Hospital, BHU. This is a tertiary care teaching hospital, which serves the population of eastern region of Uttar Pradesh and adjoining districts of Bihar, Jharkhand and Madhya Pradesh, India.

### Media and Culture Conditions

All clinical samples except urine were first inoculated onto blood agar (Hi-Media, India) and MacConkey agar (Hi-Media, India) plates whereas the urine samples were inoculated only on CLED agar (Hi-Media, India) plates. The plates were incubated at 37°C for 24–48 h. The identification of isolates was done according to standard method described elsewhere [19].

### Growth on Mannitol Salt Agar

All staphylococcal isolates were again inoculated onto mannitol salt agar (Hi-Media, India) and plates were incubated at 37°C for 24–48 h. Mannitol fermentation was observed and recorded.

### Coagulase Test

Slide coagulase tests of all 1681 isolates were performed by emulsifying few pure colonies of staphylococci from blood agar on undiluted rabbit plasma. Tube coagulase tests were performed by diluting the plasma in freshly prepared normal saline (1:6). Three to four pure colonies were emulsified in 1 ml of diluted plasma and the tubes were incubated at 37°C. Readings were taken at 1 h, 2 h, 3 h, and 4 h and further incubated overnight at room temperature if no clot formation was observed. *S. aureus *ATCC 29213 was used as control strain [19].

### Latex agglutination test (Slidex Staph Plus)

Latex agglutination test on all clinical isolates was performed according to the protocols supplied by the manufacturer (Biomerurix India Ltd, New Delhi, India).

### Determination of Minimum Inhibitory Concentration (MIC)

MIC of oxacillin (Hi-Media, India), vancomycin (Lilly Pharma, Giessen, Germany) and teicoplanin (Gruppo Lepetit, Angani, Italy) were determined by agar dilution method as described elsewhere [20]. Briefly, gradient plates of Mueller-Hinton agar (Hi-Media, India) were prepared with oxacillin (0.25–256 μg/ml) (with 2% NaCl), vancomycin (0.5–128 μg/ml) and teicoplanin (0.5–128 μg/ml). By direct colony suspension method 0.5 McFarland equivalent inouculum were prepared in normal saline from 18–24 h agar plate culture. The suspension was further diluted to achieve desired inoculum concentration of 10^5 ^CFU/ml. All strains were spotted onto gradient plates. Plates were incubated overnight at 35°C for any visible growth. Readings were taken according to NCCLS guidelines [20]. *S. aureus *ATCC 29213 and *Enterococcus faecalis *ATCC 29212 strains were used as vancomycin susceptible controls and *E. faecalis *ATCC 51299 as vancomycin resistant control.

### Inoculation on BHI vancomycin screen agar

In-house prepared BHI agar (Hi-Media, India) screen plates containing 6 μg/ml vancomycin (Lilly Pharma, Giessen, Germany) were prepared. Inoculum suspensions were prepared by selecting colonies from overnight growth on nutrient agar plates. The colonies were transferred to sterile saline to produce a suspension that matches the turbidity of a 0.5 McFarland standard. The final inoculum concentration of 10^5 ^to 10^6 ^CFU per spot was prepared by adding the sterile saline to the bacterial suspension. These suspensions were inoculated onto BHI screen agar plates and were incubated for 24 h at 35°C in ambient air. Any visible growth indicated the vancomycin resistance. *S. aureus *ATCC 29213 and *Enterococcus faecalis *ATCC 29212 strains were used as a vancomycin susceptible control strains and *E. faecalis *ATCC 51299 as vancomycin resistant control strain.

### Disc Diffusion

Disc diffusion test of Penicillin (10 U), Oxacillin (1 μg), Gentamicin (10 μg), Tobramycin (30 μg), Amikacin (30 μg), Netilmicin (30 μg), Norfloxacin (10 μg), Ciprofloxacin (5 μg), Chloramphenicol (30 μg), Erythromycin (15 μg), Tetracycline (15 μg), Trimethoprim/Sulfamethaxole (1.25/23.75 μg), Nitrofurantoin (300 μg), Vancomycin (30 μg), Cefoperazoneoxacillin/Sulbactam (75/30 μg) was carried out using Kirby-Bauer Method as described elsewhere [20]. Mueller-Hinton agar plates were overlaid with the inoculum (turbidity equivalent to that of a 0.5 McFarland Standard) of the *S. aureus *clinical strains. Zone diameters were measured at 24 and 48 h following NCCLS criteria [20]. *S. aureus *ATCC 29213 was used as reference strain.

### Detection of *mecA *gene by PCR

Staphylococcal DNA was isolated using classical chloroform: phenol extraction method described elsewhere [21]. The oligonucleotide primers for *mecA *gene (mecA F 5' GTAGAAATGACTGAACGTCCGATGA 3' and *mecA *R 5' CCAATTCCACATTGTTTCGGTCTAA 3') and reaction condition previously reported were used [22]. A Biometra DNA thermocycler was programmed with the initial denaturation, 4 min at 94°C; 30 cycles with a 45-s denaturation step at 94°C, a 45-s annealing step at 56°C and a 30-s extension step at 72°C and 2 min extension step at 72°C and a holding step at 4°C until the sample was analyzed. The PCR products were electrophoresed, stained with 10 μM ethidium bromide and visualized by using UV transillumination. The positive tests have shown PCR product of 310 bp. *S. aureus *ATCC 29213, reference strain was used *mecA *positive control strain, and *S. aureus *ATCC 43300 as *mecA *negative control strain.

### Detection of *vanA *and *vanB *genes by PCR

Staphylococcal DNA was isolated as described above [21]. Oligonucleotide primers for *vanA *(*vanA *F 5'CATGAATAGAATAAAAGTTGCAATA 3'and *vanA *R 5'CCCCTTTAACGCTAATACGACGATCAA 3') and *vanB *(*vanB *F 5' GTGACAAACCGGAGGCGAGGA 3' and *vanB R*5'CCGCCATCCTCCTGCAAAAAA 3') genes and reaction condition previously reported were used [23]. A Biometra DNA thermocycler was programmed with the initial denaturation, 10 min at 94°C; 30 cycles with a 30-s denaturation step at 94°C, a 45-s annealing step at 50°C and a 30-s extension step at 72°C and 10 min extension step at 72°C and a holding step at 4°C until the sample was analyzed. The PCR products were electrophoresed, stained with 10 μM ethidium bromide and visualized by using UV transillumination. *Enterococcus faecalis *A256 and *Enterococcus faecalis *V583 were used *vanA *and *vanB *positive control strains respectively whereas *Enterococcus faecalis *ATCC 29212 was used as *vanA/B *negative control strain.

## Results

Out of 1681 staphylococcal isolates, 761 isolates were slide coagulase positive, 772 were slidex staph plus positive, and 783 were tube coagulase positive. Thus, 783 strains were confirmed as *S. aureus *and remaining 898 strains as CoNS.

MIC of 783 *S. aureus *strains and 93 CoNS against oxacillin had shown that 318 *S. aureus *stains and 51 CoNS strains were resistant to oxacillin (MIC ≥ 4 μg/ml for *S. aureus *and MIC ≥ 0.5 μg/ml for CoNS). Out of 318 oxacillin resistant *S. aureus *strains, total two strains were found to be VRSA strains. One strain had shown vancomycin and teicoplanin MIC 32 μg/ml and the other strain with vancomycin and teicoplanin MIC 64 μg/ml. Out of 51 oxacillin resistant CoNS, one strain had shown vancomycin and teicoplanin MIC 32 μg/ml. Six strains of *S. aureus *were to be found vancomycin intermediate *S. aureus *(two strains with MIC of 16 μg/ml and four strains with MIC of 8 μg/ml). Two *S. aureus *strains were to be teicoplanin intermediate (MIC 16 μg/ml). The detailed description of all vancomycin intermediate/resistant *S. aureus *strains are given in table 1.

**Table 1:** Detailed description of vancomycin resistant staphylococcal strains.

**Strains**	**No. of Isolates**	**MIC vancomycin **(μg/ml)	**MIC teicoplanin **(μg/ml)	**Specimens**	**Prior vancomycin use history**	**Ward/OPD**
S. aureus* (VRSA)	1	64	64	Pus	Yes	Surgery ward
S. aureus* (VRSA)	1	32	32	Pus	Yes	Skin ward
S. aureus** (VISA)	2	16	16	Pus	No	Orthopedic OPD
S. aureus** (VISA)	4	8	8	Pus	No	Post-operative ward
*S. epidermidis *(VRSE)	1	32	32	Tip of ETT	Yes	Intensive care unit
*S. epidermidis *(VISE)	1 1	16 16	16 16	Blood Urine	No No	Endocrinology OPD Post-operative ward

*According to NCCLS guidelines (2000) these strains have been designated as Vancomycin Resistant *Staphylococcus aureus*.

**According to NCCLS guidelines (2000) these strains have been designated as Vancomycin Intermediate *Staphylococcus aureus*.

All oxacillin resistant *S. aureus *and CoNS strains had shown presence of *mecA *gene be PCR methods. All VISA, VRSA and vancomycin intermediate and resisistant CoNS strains were grown on BHI vancomycin screen agar. Disc diffusion test revealed that all vancomycin resistant staphylococcal isolates have shown antimicrobial resistance to most of the commonly used antimicrobials (table 2). However, none of theses strains could demonstrate the presence of *vanA *and or *vanB *gene by PCR.

**Table 2:** Resistance pattern of vancomycin resistant staphylococcal isolates to commonly tested antimicrobial agents as determined by disc diffusion method.

**Strains**	**Resistant/Intermediate**	**Sensitive**
*S. aureus *(MIC van 64 μg/ml)	Pen, Oxa, Gen, Tob, Amik, Net, Nor Cip, Chl, Ery, Tri/Sul, Cefo/Sul, Van	Tet, Nit
*S. aureus *(MIC van 32 μg/ml)	Pen, Oxa, Gen, Tob, Amik, Net, Nor, Cip, Chl, Ery, Tri/Sul, Nit, Van	Tet, Cefo/Sul
*S. aureus*(MIC van 16 μg/ml)	Pen, Oxa, Gen, Tob, Amik, Net, Nit, Nor, Cip, Ery, Van	Chl, Tri/Sul, Cefo/Sul
*S. aureus*(MIC van 8 μg/ml)	Pen, Oxa, Gen, Tob, Amik, Net, Nor, Cip, Ery, Van	Tri/Sul, Nit, Cefo/Sul
*S. epidermidis *(MIC van 32 μg/ml)	Pen, Oxa, Gen, Tob, Amik, Net, Nor, Cip, Chl, Ery, Cefo/Sul, Nit, Van	Tri/Sul
*S. epidermidis *(MIC van 16 μg/ml)	Pen, Oxa, Gen, Tob, Net, Nor, Cip, Ery, Van	Tri/Sul, Nit, Amik, Cefo/Sul

Pen: Penicillin, Oxa: Oxacillin, Gen: Gentamicin, Tob: Tobramycin, Amik: Amikacin, Net: Netilmicin, Nor: Norfloxacin, Cip: Ciprofloxacin, Chl: Chloramphenicol, Ery: Erythromycin, Tet: Tetracycline, Nit: Nitrofurantoin Tri/Sul: Trimethoprim/Sulfamethaxole, Cefo/Sul: Cefoperazoneoxacillin/Sulbactam, Van: Vancomycin.

## Discussion

In the routine microbiology laboratory prompt identification of the staphylococcal strains up to species level were done by catalase, coagulase and other standard biochemical test. However during routine screening by slide coagulase test many strains of *S. aureus *are missed due to their poor sensitivity and falsely reported as coagulase negative staphylococci during routine screening process. In our study we performed tube coagulase tests of all 1681 staphylococcal strains. Therefore the main criterion used for the *S. aureus *identification was tube coagulase test. However, the slidex staph plus test was also found to be good to differentiate *S. aureus *and CoNS.

MICs of oxacillin, vancomycin and teicoplanin against *S. aureus *and CoNS revealed that there is a rise in the resistance. *S. aureus *and CoNS have shown 40.61 % and 54.83 % oxacillin resistance, respectively. We have found two MRSA strains which were showing vancomycin resistance (VRSA). These VRSA strains have been isolated from the pus of two different patients who were admitted in the different wards. One VRSA (vancomycin MIC 64 μg/ml) strain was isolated from pus of a 56 years old patient who was admitted in Medicine ward and; the other VRSA (vancomycin MIC 32 μg/ml) strain was isolated from pus of a 49 years old patient who was admitted in Skin and VD ward of our hospital (table 1). Both the isolates were also found to be resisistant to several other antimicrobials such as gentamicin, tobramycin, amikacin, norfloxacin, ciprofloxacin, erythromycin, tetracycline, trimethoprim/sulfamethoxazole and cefoperazone/sulbactam (table 2). While going through the detailed history of these two patients it was found that they were already treated with glycopeptides for more than 20 days. These patients were not responding to further vancomycin therapy and later died due to complication of the disease.

The present study also encountered one vancomycin resistant CoNS strain with vancomycin and teicoplanin MIC 32 μg/ml. The strain was later identified as *Staphylococcus epidermidis*. This strain was isolated from culture of tip of endo-trachial tube of a patient who was admitted in the intensive care unit of teaching hospital.

Of total six VISA strains, four had their MIC 8 μg/ml and two strains showed vancomycin and teicoplanin MIC of 16 μg/ml. Out of six VISA strains, two were isolated from the pus specimens of the patient visiting outpatient department and remaining four were from the pus specimens of patients admitted in post-operative ward.

Out of two strains of CoNS (*Staphylococcus epidermidis*) with vancomycin and teicoplanin MIC 16 μg/ml, one was isolated from the blood of a patient who was admitted in orthopedic ward, and the other was isolated from the urine specimen of a patient visiting the endocrinology outpatient department.

In this study we have found almost similar pattern of MIC of vancomycin and teicoplanin. The present study shows that there is a significant rise of reduced susceptibility of oxacillin, vancomycin and teicoplanin. The emergence of the glycopeptide resistance is of great concern. Though first case of VRSA was reported in 2002 in USA [9] but few other countries have reported the reduced susceptibility of *S. aureus *and CoNS against glycopeptides [6-8,10,11,13-18]. Recently Pallazo et al [10] have also reported some vancomycin resistant strains of CoNS in Brazil. More recently Bathaineh has reported VRSA strains from Jordan [11]. Ashdulla et al [18] have reported some strains of vancomycin intermediate *S. aureus *(VISA) from India. Song et al, 2004 have also been reported the emergence of heterogeneous vancomycin resistant *S. aureus *strains from India and its neighboring countries [24]. The current vancomycin resistant staphylococci in hospital as well as in community are alarming situation to the clinicians. The development of antibiotic resistance in developing countries like ours seems to be very much related to the irrational antibiotic usage due to it's easy availability at the drug store without prescription, injudicious use in hospitals and uncontrolled use in agriculture, animal husbandry and fisheries [25]. This emergence of VRSA/VISA may be due to building of selective pressure of vancomycin. Vancomycin, a glycopeptide is currently the main antimicrobial agent available to treat life-threatening infections with MRSA. Until recently vancomycin resistance among gram-positive bacteria had been thought to be uncommon but the confirmed reports of vancomycin resistance in *Enterococcus *spp; *S. aureus *and CoNS have been reported from various part of world. Widespread use of vancomycin to treat infections caused by MRSA and other gram-positive cocci has led to the emergence of vancomycin resistance. The large scale of development and subsequent spread of resistance to vancomycin has been perceived as a fearsome threat to the already challenging therapy of MRSA.

The true mechanism of vancomycin resistance in *S. aureus *is not known. It was initially feared that *S. aureus *would acquire the *van *gene that code for vancomycin resistance in *Enterococcus s*pp; this phenomenon was successfully accomplished in the laboratory [26]. Further, Showsh et al, 2001 [27] have demonstrated the presence of sex pheromone in *S. aureus *that promotes plasmid transfer in *Enterococcus *spp. Release of these pheromones by *S. aureus *with proximity to vancomycin-resistant enterococci causes the transfer of plasmids encoding *van *gene to the *S. aureus*.

In current study all strains of VRSA and VISA strains were negative for *vanA/vanB *gene by PCR. Therefore the absence of *vanA/B *genes in the present isolates dose not rule out that these strains are not VRSA or VISA. There is another hypothesis which says that cell wall thickening is responsible for the development of vancomycin resistance. The mechanism of vancomycin resistance has been extensively studied with the first clinical VRSA strain, Mu50 [28-30]. Biochemical and transmission electron microscopy (TEM) examination of the Mu50 cell, suggested that it produces increased amounts of peptidoglycan. More murein monomers and more layers (probably 30–40 layers as judged by cell-wall thickness observed with TEM) of peptidoglycan are considered to be present in the cell wall. As a result, more vancomycin molecules are trapped in the peptidoglycan layers before reaching the cytoplasmic membrane where peptidoglycan synthesis occurs. Moreover, a higher concentration of vancomycin would be required to saturate all the murein monomers that are supplied at an increased rate in Mu50. Besides the vancomycin-trapping mechanism, designated "affinity trapping," [31-33], Hiramatsu has suggested that dense accumulation of vancomycin molecules within the thickened cell-wall significantly delays the timing of complete inhibition of cell-wall synthesis by not allowing efficient penetration of vancomycin molecules through the thickened cell-wall layers [30].

The thickened cell wall of VRSA strains become thinner with the loss of vancomycin resistance during the drug free passage and again become thick in resistant mutants. Pallazo et al [10] have also demonstrated the thickening of cell wall in vancomycin resistant staphylococci. This could be the possible mechanism behind the vancomycin resistant staphylococcal isolates that we have reported though we could not perform the test for the demonstration of cell wall thickening in these isolates.

## Conclusion

Our study for the first time has exposed the emergence of VRSA and VISA in North India. Although we confined our study within North India, the emergence of VRSA/VISA might also be prevalent in other parts of India as antibiotic misuse is equally common there. Hence, there should be an immediate response from the concerned authorities to check further emergence and spreading of these notorious VRSA strains. A strict regulation on irrational antibiotic usages might be an appropriate and effective approach in this direction. Moreover, nationwide surveillance program should be carried out to map the vancomycin susceptibility pattern in this country. For this, all strains with vancomycin MIC 4 μg/ml should be earmarked and sent to the reference laboratory for the further characterization. This will help to identify the potential areas which are already under the major threat of VRSA/VISA emergence and therefore draw more focused attention of Government for prompt tackling of this problem.

## Competing interests

The author(s) declare that they have no competing interests.

## Authors' contributions

HK has conceived and designed the work and performed strain isolation and identification, antibiotic susceptibility testing, PCR tests and prepared the manuscript. MR has participated in the designing of the work and correction of manuscript. Both the authors read and approved the final manuscript.

## Pre-publication history

The pre-publication history for this paper can be accessed here:


